# Parent-Child Interactions May Help to Explain Relations Between Parent Characteristics and Clinically Observed Child Autistic Behaviours

**DOI:** 10.1007/s10803-023-05914-x

**Published:** 2023-05-20

**Authors:** Antonina Loncarevic, Murray T. Maybery, Josephine Barbaro, Cheryl Dissanayake, Jonathan Green, Kristelle Hudry, Teresa Iacono, Vicky Slonims, Kandice J. Varcin, Ming Wai Wan, John Wray, Andrew J. O. Whitehouse

**Affiliations:** 1 CliniKids, Telethon Kids Institute, Nedlands, WA Australia; 2 School of Psychological Science, University of Western Australia, Crawley, WA Australia; 3 Cooperative Research Centre for Living with Autism, Long Pocket, Indooroopilly, QLD Australia; 4 Olga Tennison Autism Research Centre, School of Psychology and Public Health, La Trobe University, Bundoora, VIC Australia; 5 Division of Neuroscience and Experimental Psychology, School of Biological Sciences, University of Manchester, Manchester, UK; 6 Manchester Academic Health Science Centre, Manchester University NHS Foundation Trust, Greater Manchester Mental Health NHS Trust, Manchester, UK; 7 Department of Psychology and Counselling, School of Psychology and Public Health, La Trobe University, Bundoora, VIC Australia; 8 Living with Disability Research Centre, College of Science, Health, and Engineering, Victoria, Australia; 9 Children’s Neurosciences, Institute of Psychiatry, Psychology and Neuroscience, Evelina London Children’s Hospital, Kings College London, London, UK; 10 School of Allied Health Sciences, Griffith University, Gold Coast, Brisbane, QLD Australia; 11 Perinatal Mental Health and Parenting Research Unit, Division of Psychology and Mental Health, School of Health Sciences, University of Manchester, Manchester, UK; 12 Child and Adolescent Health Service, Child Development Service, West Perth, WA Australia; 13 University of Western Australia, Crawley, WA Australia

**Keywords:** Autism, Parents, Broader autism phenotype, Psychological distress, Parent-child interaction, Mediation

## Abstract

The importance of supporting parent-child interactions has been noted in the context of prodromal autism, but little consideration has been given to the possible contributing role of parental characteristics, such as psychological distress. This cross-sectional study tested models in which parent-child interaction variables mediated relations between parent characteristics and child autistic behaviour in a sample of families whose infant demonstrated early signs of autism (N = 103). The findings suggest that associations between parent characteristics (psychological distress; aloofness) and child autistic behaviours may be mediated by the child’s inattentiveness or negative affect during interactions. These findings have important implications in developing and implementing interventions in infancy which target the synchrony of parent-child interaction with the goal to support children’s social communication development.

Autism Spectrum Disorder (autism) is a neurodevelopmental condition marked by difficulties with social communication and interaction, and by restricted, repetitive behaviours and interests. Autism is highly heritable, with estimates ranging from 56 to 95% (Colvert et al., 2015). Increasingly, it is thought that differences in early brain development affect later phenotypic outcomes in children with autism (Johnson et al., 2021; Klin et al., 2020). Social engagement has been identified as a significant moderator between early brain differences and behavioural phenotypes, such as autism (Johnson et al., 2021), and a particular focus has been placed on the quality of a child’s social interactions. Interactive specialization theory proposes that the quality of a child’s early social interactions influences the development of brain structures that underpin social behaviour (Johnson, 2011). It is argued that early parent-child interactions are a key aspect of the child’s early social environment and are, therefore, important in shaping an optimal social environment for the development of neural pathways related to social behaviours. In line with this theory are findings that sensitive and responsive parental interaction styles are associated with more optimal social and communicative outcomes for children experiencing typical (Madigan et al., 2019; Tamis-LeMonda et al., 2001) and atypical development (Baker et al., 2010; Siller & Sigman, 2002).

In the context of autism, increasing the quality of parent-child interactions has become a key target in pre-emptive parent-mediated interventions designed to support more optimal developmental outcomes for infants at high likelihood of autism. In this regard, it is not hypothesized that parental interactions are a ‘cause’ of autism, but rather that atypical social cues early in life may contribute to differences in parent interaction styles, which then modifies the quality of the social input the infant receives. A randomised controlled trial (RCT) of 54 infant siblings of autistic children aged 7 to 10 months considered at higher likelihood of developing autism (through sibship with a child with autism) found that parent-mediated video-aided intervention (iBASIS-VIPP) significantly reduced parental directiveness as well as autism symptomatology at intervention endpoint after 5 months of treatment (Green et al., 2015) and these gains were mostly retained at follow-up 24 months post-intervention (Green et al., 2017). A more recent RCT of 103 infants aged 9 to 14 months demonstrating early signs of autism showed no effects of iBASIS-VIPP on parental directiveness and responsiveness during parent-child interactions immediately post 5 months of intervention (Whitehouse et al., 2019). However, a longitudinal follow-up of the same cohort demonstrated improvement in parent responsiveness and a reduction in the severity of autism symptoms across early childhood resulting in reducing odds of an autism diagnosis at 3 years of age for those children who received the intervention (Whitehouse et al., 2021). While these findings are promising in improving parent-child interaction and reducing autism symptom severity, these studies generally do not consider important factors, such as parent characteristics, that may affect parent-child interaction dynamics.

Research has so far identified associations across two of the following domains: parent characteristics such as parental phenotypic behaviours and mental health, parent-child interaction, and child autistic behaviour, and this work will be outlined in the following sections. However, no studies have investigated models linking all of these domains. Given the associations between these domains, it is imperative to examine possible causal relations, which may facilitate the prioritising of families into interventions that are specifically tailored to target the needs of both parent and child to ensure optimal outcomes. Given that many early interventions rely on parents to deliver the intervention, it is essential that we support the parents by encouraging their strengths and providing specific supports in areas where they might be experiencing difficulties.

## Associations Between Parent Characteristics and Child Outcomes

One line of research has identified links between parent characteristics, such as the broader autism phenotype (BAP), and child outcomes. The BAP refers to subtle expressions of social communication behaviours and/or restricted interests that do not meet the threshold for autism clinical diagnosis (Hurley et al., 2007; Losh & Piven, 2007). Research shows that more pronounced BAP traits in parents have been associated with more autism-related behaviours in their children (Sasson et al., 2013). Also, children with at least one parent with more pronounced BAP traits were reported to have poorer structural and pragmatic language outcomes than children of parents with lower BAP traits (Taylor et al., 2013). Furthermore, maternal BAP traits were significantly related to child social-emotional behavioural development as early as 6 months of age, and with child social, speech and symbolic skills, and repetitive behaviours at 24 months of age (Loncarevic et al., 2021).

More broadly in the general population, research shows associations between parent psychological distress and early developmental outcomes. Parent psychological distress is used as an ‘umbrella’ term to refer to the levels of either depression, anxiety, or stress in the parent. Maternal depression and anxiety during their child’s first years of life were significant predictors of child internalizing problems (Bagner et al., 2010). Furthermore, maternal depression was significantly associated with the child’s language development as early as 12 months of age (Quevedo et al., 2012). Maternal depression may also be associated with difficult child temperament, behaviour problems, and poorer cognitive and academic outcomes later in life (Wachs et al., 2009). The relations between parent stress and child behavioural problems appear to be reciprocal, particularly in families of children with developmental delays (Baker et al., 2005; Neece et al., 2012). Child behavioural problems may lead to increases in parent stress and high parent stress may lead to greater internalizing and externalizing child behavioural problems and reduced social competence (Anthony et al., 2009; Neece et al., 2012).

### Associations Between Parent Characteristics and Parent-Child Interaction

A separate body of research in developmental psychology has identified that certain parent characteristics, such as psychological distress, can also influence parent interaction behaviours. Parents experiencing high levels of psychological distress are more likely to be less responsive and less sensitive in their interactions with their children (Bayer et al., 2006; Flykt et al., 2009; Wachs et al., 2009). Xu et al. (2005) described that mothers with high levels of psychological distress demonstrated less sensitivity in parent-child interactions. They concluded that these mothers may be lacking the psychological resources to be sensitive to their child’s needs, or they may feel helpless or incompetent when interacting with their child possibly due to the negative cognitive bias associated with psychological distress. Furthermore, parent depression, stress, and anxiety, have been found to affect parental sensitivity (Shin et al., 2008) and also parental emotional availability in families with a child with cerebral palsy (Barfoot et al., 2017). Therefore, higher levels of parent psychological distress appear to impact parent interaction behaviours in parent-child interactions.

### Associations Between Parent-Child Interaction and Child Outcomes

Another body of research has focused on investigating the quality of parent-child interactions in relation to autism. Two studies by Wan et al. (2012, 2013) reported differences in parent-child interactions for children with a higher likelihood of developing autism (through sibship with a child with autism) compared to children with no family history of autism. When the children were 6 and 12 months of age, parents in the higher likelihood group showed less sensitivity to their child’s behavioural cues and greater directiveness during play compared to parents in the lower likelihood group (Wan et al., 2012). Furthermore, at 6 months, infant liveliness was lower in the higher likelihood group, and at 12 months, infant attentiveness to caregiver, positive affect, and dyadic mutuality and intensity of engagement were all significantly lower in the higher likelihood group (Wan et al., 2013). The differences in dyadic mutuality, infant positive affect and attentiveness predicted autism status at age 3 years (Wan et al., 2013). The findings do not imply that the parents are in any way a ‘cause’ of autism; rather they suggest that atypical communication and social cues among the children may induce differences in parent interaction behaviours, which in turn, may modify the quality of social input that the child receives. These reciprocal influences between parent and child are supported by findings suggesting that within the higher likelihood group, parents adapt their behaviour to the specific abilities of their child (Bontinck et al., 2018). Furthermore, a recent systematic review suggested that there are other infant communicative differences between the higher and lower likelihood groups, such as gesture, which may impact on how parents interact with their infants (Wan et al., 2019).

### The Present Study

The aim of the present study was to examine the relations between parent characteristics (BAP, psychological distress), parent-child interaction and child outcomes, and to investigate whether specific qualities of the parent-child interaction may mediate the relations between parent characteristics and child autism-related outcomes. We investigated these associations in a cohort of children showing early signs of autism. Past research has typically focused on examining associations between parent characteristics and child outcomes in cohorts of children at elevated genetic risk of autism as defined by having an autistic sibling. However, only approximately one fifth of these children at elevated risk go on to receive an autism diagnosis (Ozonoff et al., 2011), and the genetic and environmental factors influencing their development may be different to the influential factors for the broader autism population (Dissanayake et al., 2019). The children included in this study were identified through a community clinical pathway and so may be more likely to show broader developmental concerns, including behaviours indicative of autism, than the sibling cohorts.

Based on previous findings, we expected that higher levels of BAP traits in parents, often characterised by subtle autism-like social communicative behaviours, would disrupt reciprocal parent-child interactive dynamics in ways that usually support and facilitate social-communicative development. This may have cascading effects on autism-related atypicalities of which social communication is a major component. Furthermore, we predicted that higher levels of psychological distress would influence how parents interact with their child, potentially leading to greater display of autistic behaviours in the child. Therefore, we hypothesized that higher levels of parent BAP traits and psychological distress would be related to child outcomes, particularly higher levels of autistic traits, and these effects would be mediated by parent-child interaction.

## Methods

### Participants

One hundred and three families were recruited as part of the Australian Infant Communication and Engagement Study (AICES) conducted in Perth, Western Australia and Melbourne, Victoria (Whitehouse et al., 2019). AICES is a single-blind RCT testing the effectiveness of the iBASIS-VIPP parent-mediated early intervention in a cohort of young children (aged 9–14 months) displaying early signs of autism. In Perth, families were recruited through referrals to the Government metropolitan service for children with developmental delays. In Melbourne, families were directly referred to the study team by community-based maternal and child health nurses. Following referral, the child was administered the 12-month checklist of the Social Attention and Communication Surveillance-Revised (SACS-R; Barbaro & Dissanayake 2010, 2013; Mozolic-Staunton et al., 2020) tool. Due to the different recruitment pathways between sites, the eligibility screen was completed with each child’s caregiver over the phone by a study team member. For families to be eligible for the study, the young child had to have “atypical” responses on at least three of the five ‘key’ SACS-R behaviours most predictive of a diagnosis of autism (Barbaro & Dissanayake, 2013). The five key behaviours on the 12-month SACS-R checklist are atypical responses relating to: spontaneous eye contact, proto-declarative pointing, social gestures, imitation, and response to name. The SACS-R is an assessment tool devised to assess early autism signs during routine child health checks. In AICES, all items on the SACS-R required the child to engage in an action and it was noted by the clinician whether the child responds with a “typical” or “atypical” behaviour. For example, the item measuring imitation states: “Get the child’s attention. Use a brush/comb on your hair. Give it to the child and say ‘your turn’. Does s/he imitate you?” At 12 months of age, the SACS-R has an estimated positive predictive value of 72% for subsequent diagnosis of autism (Barbaro et al., 2018).

### Procedures

This study draws on the data collected at the baseline assessment during the AICES RCT. Baseline assessments took place within 4 weeks of the eligibility screening and consisted of a combination of parents completing questionnaires on themselves and their children and behavioural assessments completed at the Telethon Kids Institute (Perth) and La Trobe University (Melbourne) by blinded assessors who were part of the study team. One hundred families included two parents, with the mother completing all the parent-report measures of the child’s developmental outcomes and also the self-report measures. For these families, the mother also participated in the play activities that were coded for parent-child interaction. As the current study was designed to assess the relations between parent characteristics and parent-child interaction, it was necessary that parent variables be available for the parent participating in the interaction. This meant that the data from three families where the father completed the parent-child interaction, but the mother completed the questionnaires could not be used. As a result, the final sample consisted of 100 mother-child dyads and mothers are the parent referred to in the following sections.

## Measures

### Parent Characteristics

#### Broader Autism Phenotype Quotient (BAPQ; Hurley et al., 2007)

The BAPQ was used to assess broader autistic traits in the domains of aloof personality (lack of enjoyment or interest in social interaction), rigid personality (difficulty adjusting to change or lack of interest in change) and pragmatic language issues (difficulties with the social aspects of language). The BAPQ consists of 36 self-report items rated on a 6-point Likert scale ranging from 1 = “very rarely” to 6 = “very often”. Examples of items are: “I would rather talk to people to get information than to socialize” (aloof personality), “People have to talk me into trying something new” (rigid personality), and “It’s hard for me to avoid getting sidetracked in conversation” (pragmatic language issues). The BAPQ has demonstrated good internal consistency, with Cronbach α ranging from 0.85 for the pragmatic language subscale to 0.95 across all 36 items; and high sensitivity and specificity in differentiating between individuals with and without clinical evidence of the BAP (Hurley et al., 2007).

#### Depression, Anxiety, Stress Scales (DASS-21; Lovibond & Lovibond, 1995)

The DASS-21 was used to measure the parent’s self-reported psychological distress. The questionnaire includes subscales for depression, anxiety, and stress, with 7 items per subscale. Example items are: “I couldn’t seem to experience any positive feeling at all” (depression), “I experienced breathing difficulty (e.g., excessively rapid breathing, breathlessness in the absence of physical exertion)” (anxiety), and “I found myself getting agitated” (stress). Each item is rated on a 4-point Likert scale ranging from 0 = “did not apply to me at all” to 3 = “applied to me very much or most of the time”. Higher scores indicate greater psychological distress. Investigations of reliability and validity of DASS-21 have demonstrated good internal consistency, with Cronbach α ranging from 0.82 for the anxiety scale to 0.93 for the total scale score (Henry & Crawford, 2005); good convergent and discriminant validity (Henry & Crawford, 2005); and acceptable concurrent validity (Crawford & Henry, 2003). In the current data set the three subscale scores were highly intercorrelated (range *r* = .46 to *r* = .73), forming a single factor in a principal components analysis, and so the DASS-21 total score was used as a measure of psychological distress in the parent (Chetcuti et al., 2020).

### Child Outcomes

#### Autism Observation Scale for Infants (AOSI; Bryson et al., 2008)

The AOSI is a play-based assessment with standardized instructions designed to measure early behavioural signs associated with autism. Some examples of these early behavioural signs are social reciprocity, response to name, and atypical motor and sensory behaviours. At each testing site, a blinded assessor trained to fidelity administered the AOSI. The AICES RCT used the 18-item version of the AOSI, which includes 16 scoring items that are coded from 0 to 2 or 3, yielding a total score with a maximum value of 38. Higher scores indicate greater expression of autism symptomatology. The AOSI has demonstrated strong inter-rater reliability based on Cohen’s κ of 0.92 (Bryson et al., 2008) and moderate accuracy in predicting autism diagnostic status at 36 months when administered to infant siblings of autistic children at 14 months of age (Bussu et al., 2018). All of the AOSI assessments were filmed, and the assessor coded the video recordings. 20% of videos were selected at random to also be coded by the assessor at the other study site to measure inter-rater agreement. This double coding showed good inter-rater agreement (ICC = 0.78). Furthermore, a previous study has demonstrated strong inter-rater agreement in video-coded AOSI total scores (Hudry et al., 2021).

#### Infant-Toddler Social Emotional Assessment (ITSEA; Carter et al., 2003)

The ITSEA, a 170-item parent-report questionnaire, was used to measure social-emotional and behavioural problems in children. The items are rated on the following scale: 0 = “not true/rarely”, 1 = “somewhat true/ sometimes”, and 2 = “very true/often”. The ITSEA assesses four domains of behaviour - externalizing, internalizing, dysregulation, and competencies; and three clusters of more serious problems – maladaptive, atypical behaviour, and social relatedness. Atypical behaviour and social relatedness clusters assess behaviours that may be indicative of autism and are, therefore, selected for the analyses in this study. Example items on these two clusters are: “puts things in a special order over and over and gets upset if he or she is interrupted” (atypical behaviour), and “looks right at you when you say his or her name” (social relatedness). This measure was found to have good reliability and validity. Cronbach α for internal consistency of the ITSEA range from 0.85 to 0.90; test-retest reliability coefficients range between 0.76 and 0.91; and this measure has demonstrated convergent and divergent validity as well as sensitivity and specificity (Carter et al., 2003; Gokiert et al., 2014).

### Parent-Child Interaction

#### Manchester Assessment of Caregiver-Child Interaction (MACI; Wan et al., 2017)

The MACI was used to measure the quality of the parent-child interaction. For the purposes of the MACI, parents were asked to engage in free play with their child for 6 minutes and their interaction was video recorded. The interaction took place in a standardised research setting using standardised MACI toys. The parent was instructed to commence free play on a floor matt and to let the play develop naturally as it would at home. A blind assessor trained to fidelity rated each of the 8 scales of the MACI on a 7-point scale, ranging from 1 to 7, and the total duration of the recording was coded. The 8 scales of the MACI are: caregiver sensitive responsiveness (appropriate, timely responding to child behaviour with the aim of meeting the child’s immediate, interactive, and developmental needs; appropriate engagement, support and structuring, warmth, and an attentive attitude), caregiver nondirectiveness (focus on the child’s experience, rather than using directiveness and placing demands on the child through demanding and intrusive behaviours, and negative comments), child attentiveness to caregiver (interest in the caregiver through direct eye contact or joint activity, face/body orientation acceptance of and interest in caregiver, and other references to caregiver activity, such as imitation), child liveliness (amount and level of spontaneous physical activity), child positive affect (amount and degree of positive affect expressed through behaviour and vocalization), child negative affect (amount and degree of negative affect expressed through bodily gestures and vocalization), dyad mutuality (amount and level of reciprocity, attunement and “togetherness”), and dyad engagement intensity (degree of intensity of engagement by both parties, including the degree of interest, arousal and positivity/excitement). An independent trainer double coded 15% of the recordings, which yielded good to high inter-rater agreements; single measures ICC, two-way mixed effects model range 0.67-0.80.

### Data Analysis

We examined three sets of associations, those between parent and child variables, parent and parent-child interaction variables, and parent-child interaction and child variables. Pearson correlations assessed these associations. To examine whether the relations between parent and child variables are mediated by parent-child interaction, mediation analyses were performed using the PROCESS macro for SPSS (Hayes, 2018).To test mediation, significant correlations between the mediator and both the independent and dependent variable is a necessary precondition (Hayes, 2018). To meet this precondition, parent variables needed to be significantly correlated to parent-child interaction variables and child variables also needed to be significantly correlated to the same parent-child interaction variables as the parent variables. An alpha level of 0.05 was used to test for statistical significance.

## Results

### Data Screening and Descriptive Statistics

The data were screened for outliers and any score that was more than 2.2 times the Interquartile Range (IQR) from the mean was considered an outlier (Hoaglin & Iglewicz, 1987). All such outliers (n = 8) were replaced with the next most extreme score that was not considered to be an outlier (see Field 2013). Multivariate outliers were also examined, and none were found using Mahalanobis distance (Cabana et al., 2019). After dealing with the outliers in the data, all skew and kurtosis values indicated that the data were appropriate for parametric analysis. Skew was less than |2.0| and kurtosis was less than |9.0| (West et al., 1995).

The final sample consisted of 100 families in which there were 67 male children and 33 female children. Maternal age ranged from 22 to 43 years (*M* = 33.91, *SD* = 4.52). Descriptive statistics on all measures are shown in Table 1. Of note are the mean scores for the AOSI and the ITSEA clusters, which are all close to the cut-off scores used to flag autism concern in samples of infants who later develop autism. A total score of 9 on the AOSI and scores of 0.60 on ITSEA atypical behaviour and 1.31 on ITSEA social relatedness are used as cut-off scores of concern. Therefore, the means on these measures indicate that the sample was at an elevated likelihood of developing autism.

**Table 1:** Descriptive Statistics

	*n* data		Range	*M*	*SD*
Child age (months)		100	9.07–16.33	12.32	1.96
**Child Measures**					
AOSI		100	1–26	9.59	4.25
ITSEA Atypical Behaviour		91	0–1.40	0.59	0.29
ITSEA Social Relatedness		91	0.20 − 1.90	1.31	0.38
**Parent Measures**					
BAPQ Aloof		97	1.08–4.17	2.65	0.69
BAPQ Pragmatic Language		97	1.17–4.08	2.34	0.57
BAPQ Rigidity		97	1.42–4.50	2.90	0.65
DASS-21 Psychological Distress		93	0–84	21.27	17.34
**Parent-Child Interaction Measures**					
MACI Sensitive Responsiveness		100	1–7	4.25	1.48
MACI Nondirectiveness		100	1–7	4.12	1.59
MACI Attentiveness		100	1–7	3.95	1.30
MACI Liveliness		100	2–7	4.86	1.26
MACI Positive Affect		100	1–7	3.41	1.63
MACI Negative Affect		100	1–7	2.94	1.84
MACI Mutuality		100	1–7	3.81	1.38
MACI Engagement Intensity		100	1–6	3.70	1.15

*Note.* For some of the measures *n* < 100 due to noncompletion of either child or parent questionnaires by the participants

### Associations Between Parent Characteristics and Child Outcomes

The correlations between parent characteristics and child outcomes are presented in Table 2. It is apparent here that maternal pragmatic language difficulties were associated with more pronounced atypical behaviours and reduced social relatedness in the child as measured via the ITSEA, with medium and small effect sizes, respectively. Aloofness and rigid personality traits in the mothers and their level of psychological distress, were not related to any child variable.

**Table 2:** Pearson Correlations between Child Outcome Variables and Parent Variables

	AOSI	ITSEA Atypical	ITSEA Social Relatedness
BAPQ Aloof	0.002	0.16	− 0.16
BAPQ Pragmatic Language	0.04	0.39***	− 0.28**
BAPQ Rigidity	− 0.10	0.11	− 0.09
Psychological Distress	0.05	0.19	− 0.17

* *p* < .05; ** p < .01; *** p < .001

### Associations Between Parent Characteristics and Parent-Child Interaction

We found negligible/small nonsignificant associations between parent characteristics and parent interaction behaviours (sensitive responsiveness, nondirectiveness) or dyad interaction variables (mutuality, engagement intensity), as shown in Table 3. However, some of the parent characteristics had small significant associations with child interaction behaviours (attentiveness to caregiver, liveliness, negative affect). Children whose mothers demonstrated more pragmatic language difficulties were less lively during the interaction. Furthermore, higher parent aloofness was associated with greater display of negative affect by the child during the interaction. Finally, higher levels of maternal psychological distress were related to lower child attentiveness to the parent in the interaction.

**Table 3:** Pearson Correlations between Parent Variables and Parent-Child Interaction Variables

*MACI scales*	BAPQ Aloof	BAPQ Pragmatic Language	BAPQ Rigidity	Psychological distress
**Caregiver behaviour**				
Sensitive Responsiveness	0.01	− 0.14	− 0.04	− 0.09
Nondirectiveness	0.002	− 0.14	− 0.03	− 0.17
**Infant behaviour**				
Attentiveness	0.005	− 0.11	− 0.05	− 0.26*
Liveliness	− 0.20	− 0.23*	− 0.07	− 0.04
Positive Affect	− 0.18	− 0.18	− 0.09	− 0.02
Negative Affect	0.24**	0.05	0.03	− 0.05
**Dyad behaviour**				
Mutuality	− 0.02	− 0.14	− 0.09	− 0.14
Engagement Intensity	− 0.02	− 0.04	− 0.06	− 0.09

Note. Correlation values in bold and italicized text indicate associations forming part of mediation models. * *p* < .05; ** p < .01

### Associations Between Parent-Child Interaction and Child Outcomes

We found that autistic behaviours in the child, as measured via the AOSI, were associated with (i) more directive interactions from the parent; (ii) less attentiveness to the parent and more negative affect from the child; and (iii) lower mutuality and engagement intensity in the dyadic interaction. Furthermore, atypical behaviour indicative of autism, as measured via the ITSEA, was associated with greater parent directiveness during the interaction. Finally, better social relatedness in the child was related to the child displaying more positive affect during the interaction. These correlations are presented in Table 4.

**Table 4:** Pearson Correlations between Child Outcome Variables and Parent-Child Interaction Variables

*MACI scales*	AOSI	ITSEA Atypical	ITSEA Social Relatedness
**Caregiver behaviour**			
Sensitive Responsiveness	− 0.19	− 0.17	0.08
Nondirectiveness	− 0.24*	− 0.23*	0.11
**Infant behaviour**			
Attentiveness	− 0.29**	− 0.11	− 0.06
Liveliness	0.02	− 0.20	0.01
Positive Affect	− 0.13	− 0.10	0.23*
Negative Affect	0.33***	0.09	− 0.08
**Dyad behaviour**			
Mutuality	− 0.27**	− 0.10	0.08
Engagement Intensity	− 0.22*	0.002	0.05

Note. Correlation values in bold and italicized text indicate associations forming part of mediation models. * *p* < .05; ** p < .01; *** p < .001

### Parent-Child Interaction Mediating Between Parent Characteristics and Child Outcomes

**Fig. 1:**
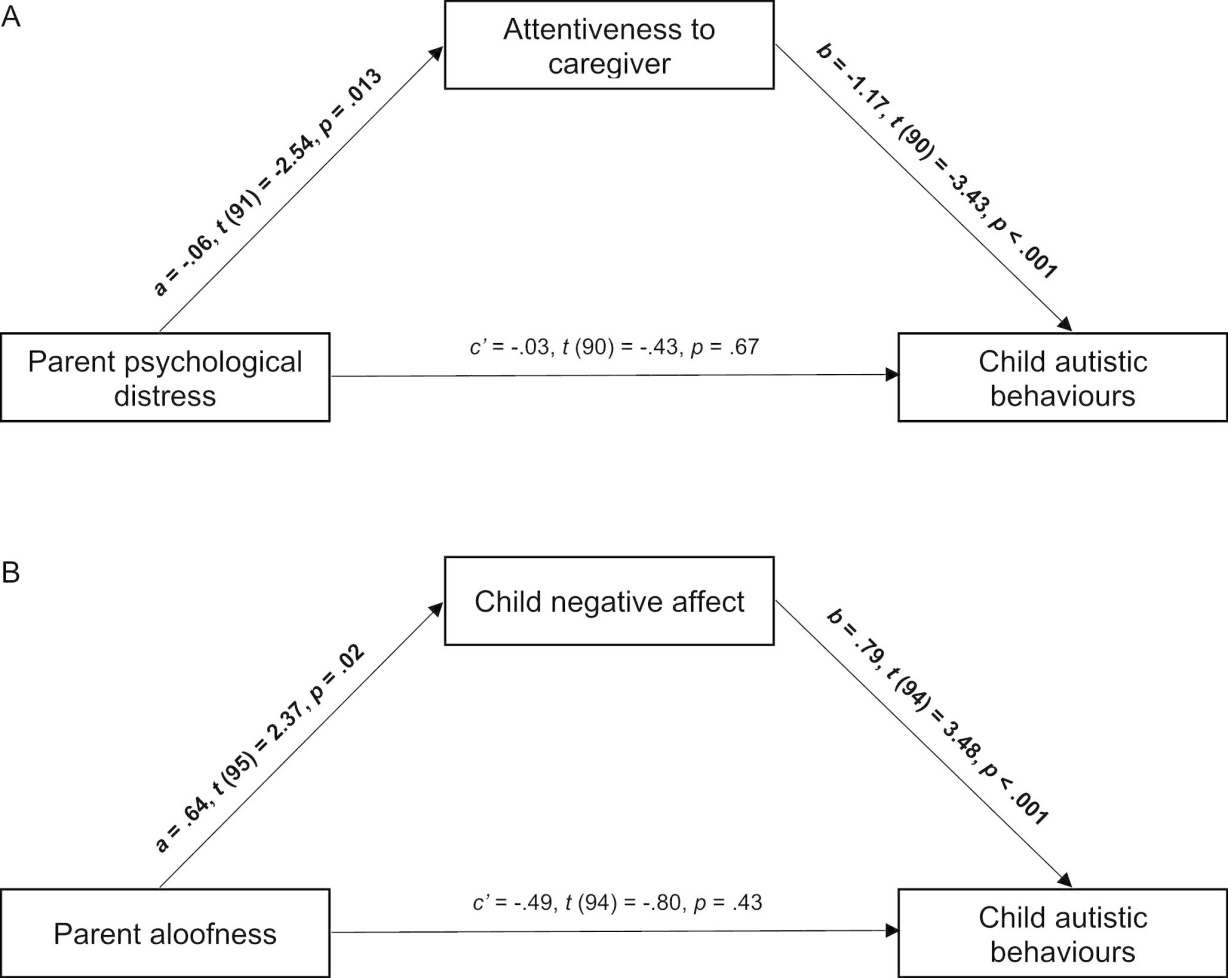
Mediation models investigating parent-child interaction variables as mediators of the relations between parent characteristics and child outcomes. Regression coefficients are unstandardized and pathways in bold are significant (*p* < .05)

Child attentiveness to caregiver, a measure of child behaviour during social interactions, correlated significantly with maternal psychological distress and also with child autistic behaviours, measured via the AOSI, suggesting a potential mediation model (Model A; see Fig. 1). Child negative affect, also a measure of the child’s interaction behaviour, correlated significantly with both maternal aloofness and child autistic behaviours, suggesting a potential second mediation model (Model B; Fig. 1). Listwise deletion of missing values was used given the sufficiently large sample size and that the data were missing completely at random. This deletion method resulted in a sample size of 93 for testing Model A and 97 for testing Model B. Both Model A and Model B accounted for a significant proportion of variance in autistic behaviours in the child (Model A: *F*(2, 93) = 5.98, *R*^*2*^ = 0.12, *p* = .004; Model B: *F*(2, 97) = 6.05, *R*^*2*^ = 0.11, *p* = .003). In both models there was no significant direct effect between the parent variables and child outcome variable, however, the mediation analyses demonstrated significant indirect effects between these variables. In Model A, there was a significant positive indirect effect between maternal psychological distress and autistic behaviours in the child through the child’s attentiveness to the caregiver (*B* = 0.0904, 95% bootstrap CI = 0.0206–0.1765). In Model B, there was a significant positive indirect effect between maternal aloofness and child autistic behaviours through the child’s negative affect (*B* = 0.0822, 95% bootstrap CI = 0.0127–0.1781).

## Discussion

The current study sought to examine whether parent-child interaction behaviours mediate the relations between parent characteristics and child outcomes in infants showing early behavioural signs of autism. We hypothesised that higher levels of parental BAP traits and psychological distress would impact on the parent’s ability to develop optimal parent-child interaction, which would in turn result in higher ratings of atypical behaviour in the child. We found that while parent psychological distress and one aspect of the BAP in the parent were each related to more pronounced autistic traits in the child, with the relations fully mediated by parent-child interaction variables, the mediators were ratings of the child’s behaviour in the interaction, rather than the parent’s behaviour as had been hypothesised. The following section discusses the mediated relations, while the subsequent sections discuss the associations between variables across pairs of the three domains (parent characteristic, parent-child interaction, and child outcomes).

### Child Interaction Variables During Parent-Child Interaction as Potential Mediators Between Parent Characteristics and Child Autistic Behaviours

Outcomes of the mediation analyses provide some support for possible causal models between parent characteristics, parent-child interaction variables, and child autistic behaviours. In particular, Model A is consistent with children paying less attention during interaction with distressed parents which may contribute to more pronounced autistic behaviours. Perhaps due to the reduced attentiveness to the parent, these children do not get as many opportunities to communicate socially with their parent, resulting in behaviours resembling those seen in autism. Likewise, in Model B, children whose mothers reported more traits of aloofness in themselves (lack of enjoyment and interest in social interaction) demonstrated more negative affect during interactions, which then related to more autistic behaviours in the child. This mediation effect might reflect the parent and child sharing a disinterest in, or even negative affect during social interaction, which could reflect heritable influences or the child learning from previous interaction experiences. Children displaying more negative affect during interactions may have fewer opportunities to develop skills in social communication, which then manifests in autistic traits. Both of these findings are important as child attentiveness and affect at 1 year have been shown to predict autism outcome at 3 years (Wan et al., 2013). As such, the mediation results may shed light on the importance of examining the contribution of parent characteristics in these relations. The mediation findings suggest that maternal aloofness and psychological distress may form part of the environmental influence on child behaviours through the child’s interaction behaviours. However, such a conclusion cannot be made with absolute certainty for both models.

In Model A, although we see a mediation effect between parent distress and child autistic behaviour through child attentiveness to caregiver, it is very possible that lower attentiveness may be due to early atypicalities seen in these children. Furthermore, these early atypicalities may be contributing to some of the parental distress. However, we did not find a direct effect between parent psychological distress and child autistic behaviours, which may support the direction of the relations captured in Model A In Model B, the direction of the relations is likely to be as proposed in the model as the parent characteristic measured, aloofness, is considered to be a stable trait (Taylor et al., 2017; Wallace et al., 2016) and, therefore, not susceptible to much influence from another’s behaviour, in this case the child’s. For both Model A and B, we also assessed the effects in reversed models (child autistic behaviour as predictor, parent-child interaction as mediator, and parent characteristic as outcome variable). While both of the reversed models yielded significant outcomes (Model A reversed – *F*(2,94) = 3.29, *R*^*2*^ = 0.07, *p* = .048; Model B reversed – *F*(2,90) = 3.13, *R*^*2*^ = 0.06, *p* = .042), the *R*^*2*^ values in the reversed models were smaller by almost 50% than the *R*^*2*^ values in the proposed Model A and Model B These findings offer some statistical support for the directionality of effects proposed by the initial mediation models, as well as that both models in both directions include considerable unexplained variance. Therefore, to assess the directionality of these effects with more certainty, future research needs to examine causal mechanisms by utilizing mediation analyses with longitudinal data.

### Associations Between Parent Characteristics and Child Outcomes

While parental aloofness and psychological distress were found to have mediated relations to child autistic behaviour, another parent characteristic was related to child behaviour without there being evidence of mediation through parent-child interaction variables. Greater maternal pragmatic language difficulties were associated with more pronounced atypical behaviours and weaker social relatedness in the child. Furthermore, we found that maternal aloofness, rigid personality traits and higher maternal psychological distress were not directly related to any child outcome variable. These findings offer some support for previous research findings. For example, Stern et al. (2017) reported that maternal pragmatic language violations were associated with weaker expressive language in autistic children and with weaker receptive language in non-autistic language-delayed children. However, few studies have examined the association between parental pragmatic language and autistic behaviour in children. Previous research has mostly focused on examining the associations between parent BAP status, based on a specific cut-off score, and child language outcomes (Bishop et al., 2006; Taylor et al., 2013). These studies reported that the presence of BAP in parents was associated with poorer structural and pragmatic language skills in the child. However, a recent study more similar in design to the present study did report that maternal BAP traits as measured on the Autism-Spectrum Quotient (Baron-Cohen et al., 2001) and maternal communication difficulties as measured via the Communication Checklist-Adult (Whitehouse & Bishop, 2009) were associated with children’s weaker social-emotional competence at 6 and 24 months of age, and also with poorer communication, symbolic abilities and social interaction, and more pronounced repetitive behaviours at 24 months of age (Loncarevic et al., 2021). Regarding the present findings that maternal psychological distress was not associated with any of the child autism-related outcomes, these results are unsurprising as previous research has shown that in samples of children with early signs of autism, maternal psychological distress was related only to child internalizing and externalizing problems and not autistic behaviours measured via the AOSI (Chetcuti et al., 2020). Additionally, the non-clinical nature of the parent sample suggests relatively low levels of psychological distress. Child outcomes may be more affected in clinically depressed and anxious samples.

Regarding the relations between maternal pragmatic language and child outcomes, this study found that greater maternal pragmatic language difficulties were associated with more pronounced atypical behaviours and reduced social relatedness in the child, a finding that diverges from past results reported by Flippin and Watson (2018) who reported no associations between maternal pragmatic language and any of the child outcomes measured in their study (child-initiated engagement and language skills). They also reported that children of mothers with higher traits of aloofness and more rigid personality traits showed weaker social engagement skills. The differences in findings could be due to the difference in the samples of the two studies. Flippin and Watson’s (2018) findings are based on a smaller sample of older children with autism as compared to the current sample of young children showing very early signs of autism. This comparison illustrates the wide variability in study designs, particularly in the samples and measures used, between the current study and past research. To our knowledge, the current study is the first to examine the association between maternal pragmatic language and autistic behaviour in very young children, and as such these results offer novel insights into the relations between these two variables.

### Associations Between Parent Characteristics and Parent-Child Interaction

In examining the mediation models, we found that higher levels of maternal aloofness were associated with the child displaying more negative affect during the interaction, and greater maternal psychological distress was related to the child’s lower attentiveness to the parent. In addition to these associations represented in the mediation models, we also found that greater levels of maternal pragmatic language difficulties were associated with less liveliness in the child during their interaction. This association is a novel finding and one that is difficult to explain. Past results suggest that liveliness might form part of a different underlying construct perhaps related to temperament rather than interaction dynamics (Wan et al., 2013). As such, the association between maternal pragmatic language and child liveliness found in the present study might be indicative of relations between this parent characteristic and child temperament. Future research is needed to examine what underlying construct liveliness taps into.

### Associations Between Parent-Child Interaction and Child Outcomes

Several direct associations emerged between parent-child interaction variables and child autistic behaviours. Greater parent directiveness during the interaction was associated with higher levels of child autistic behaviours. These associations emerged from both an objective observational measure of autistic behaviours, the AOSI, and a parent-report measure of atypical behaviours seen in autism, the ITSEA. These findings are consistent with previous findings reporting that parents of children at higher likelihood of developing autism are more directive (Wan et al., 2012). Higher parent directiveness is also reported to be more common in mothers of children with other developmental conditions, such as Down syndrome (Cielinski et al., 1995; Slonims et al., 2006). These findings do not imply in any way that parent directiveness is a cause of autistic behaviour in their child. In infants with Down syndrome, Slonims and colleagues (2006) concluded that infant behaviour was the driver for parent behaviour, whereas, in typically developing infants the parent-child interaction was somewhat affected by factors such as parental mental well-being. Therefore, it appears that it is the asynchrony in parent-child interaction which may exacerbate any underlying biological predisposition to atypical development. The transactional nature of the parent-child interaction implies that more autistic behaviours in the child might lead to parents being more directive to achieve a desired outcome in the interaction. Nonetheless, these findings indicate that interventions need to be targeting this asynchrony in parent-child interaction and helping parents develop more optimal interaction styles with their child who might be showing autistic behaviours.

In addition to higher levels of child autistic behaviours being associated with parent directiveness, children who displayed more autistic behaviours also displayed more negative affect and less attentiveness to the parent. Lastly, child autistic behaviours were related to lower mutuality and engagement intensity between the parent and the child in the interaction. In a past study, these interaction variables measured at 12 months predicted 3-year autism outcome, whereas child autistic behaviours at 12 months, as assessed by the AOSI, did not (Wan et al., 2013). Collectively, these findings of associations between autistic behaviours in children and these particular interaction variables may indicate that features of the interaction with caregivers might mediate the effect of very early behavioural atypicality on later autism diagnosis. Therefore, future research should focus on exploring the relations between these variables longitudinally and developing interventions that specifically foster more positive affect in the child, greater attentiveness to the parent, and stronger mutuality and engagement intensity.

### Limitations

This is the first study to examine correlations between the three domains of parent characteristics, parent-child interaction, and child outcomes. As such the study was exploratory in nature and no adjustments were made for multiple comparisons in testing the relations between the variables in the domains. The findings of the current study may act as a guide for future research examining the associations between these three domains and will benefit from replication in future studies. Additionally, given the cross-sectional nature of the present study, it is difficult to ascertain the directionality of relations between parent characteristics, parent-child interaction, and child outcomes. Child autistic behaviours were assessed using a direct observational measure, the AOSI, and a parent-report measure of atypical behaviours, the ITSEA, and both were associated with greater parent directiveness during the interaction. The generalizability of these relations from parent-report to clinical measures further supports findings of the transactional nature of early social interactions. It appears that children who are less socially skilled and demonstrate behaviours typically seen in autism demand their parents to be more directive during play. On the other hand, parent directiveness might limit the child from learning through experience, resulting in slower social skills development and more pronounced behaviours typically seen in autism. Mediation analyses are very powerful in examining the direction of causal relations particularly when they are applied in longitudinal study designs (Goldsmith et al., 2018; Jose, 2016; Paloma & Ricardo, 2016; Selig & Preacher, 2009). Given that the present study conducted analyses using cross-sectional data, the findings reported may not necessarily reveal the longitudinal mediation process (Shrout, 2011). Therefore, it is imperative that future research explores the causal mechanisms of the aforementioned relations in longitudinal studies.

Another limitation is the lack of behavioural measures of parental BAP traits. However, we did use a reliable self-report measure with good psychometric properties (Hurley et al., 2007; Ingersoll et al., 2011) to assess BAP traits in the parents. Furthermore, one study assessed the correlation between self-report and informant-report using the BAPQ and reported at least moderate level of self-informant agreement about the BAP (Wainer et al., 2013). Another consideration concerns the possibility that some correlations may have been influenced by shared method variance (i.e., through having the parent complete both questionnaires about their own BAP and psychological distress and also complete child measures such as the ITSEA). However, associations were found between those measures and the coding of the parent-child interaction and the other child outcome measure that was completed by assessors rather than the parent. Therefore, while shared method variance may have influenced some correlations, it is unlikely to have impacted on the full set of correlations and measures used in mediation analyses. Lastly, the AICES sample consisted of only two-parent families due to the nature of the research question, which is not reflective of all families of autistic children.

## Conclusion

The current study found that child interaction behaviours mediate relations of parental aloofness and psychological distress to autistic behaviours in the child. These findings make an important contribution to autism research concerning potential causal mechanisms in the associations between parent characteristics and child outcomes and demonstrate the importance of not overlooking the child’s contributions in social interactions. If replicated with longitudinal data, the current findings have important implications in developing and implementing interventions in very early life which target child interaction behaviours in addition to parent interaction behaviours. These interventions should aim to create an optimal synchrony in parent-child interactions which may support a child’s social communication development. While there may not be a complete consensus on what would represent optimal synchrony in parent-child interaction, there does seem to be some agreement that parent nondirectiveness and sensitive responding are key characteristics of productive parent-child interactions (Green et al., 2015, 2017; Wan et al., 2013, 2019). Therefore, past findings together with the findings of the current study inform future interventions to aim fostering these particular parent interaction behaviours, as well as child interaction behaviours to promote productive synchrony in parent-child interactions.
